# Impact of incident rheumatoid arthritis on earnings: a nationwide sibling comparison study

**DOI:** 10.1093/rheumatology/keae535

**Published:** 2024-10-16

**Authors:** Heather Miller, Martin Neovius, Johan Askling, Gustaf Bruze

**Affiliations:** Clinical Epidemiology Division, Department of Medicine Solna, Karolinska Institutet, Stockholm, Sweden; Clinical Epidemiology Division, Department of Medicine Solna, Karolinska Institutet, Stockholm, Sweden; Clinical Epidemiology Division, Department of Medicine Solna, Karolinska Institutet, Stockholm, Sweden; Clinical Epidemiology Division, Department of Medicine Solna, Karolinska Institutet, Stockholm, Sweden

**Keywords:** earnings, income, rheumatoid arthritis, register, Sweden

## Abstract

**Objectives:**

RA is known to impact work ability, but much of this knowledge comes from historical comparisons *vs* the general population that neither reflects current RA management nor distinguishes between effects of RA and pre-existing socio-economic conditions of patients. We therefore aimed to examine earnings of patients before and after RA diagnosis, using recent data and sibling comparisons.

**Methods:**

Swedish register data were used including demographic information and healthcare utilization. Participants were patients with RA (aged 30–60 years, diagnosed with RA between 2006 and 2017) identified in the Swedish National Patient Register, and their same-sex siblings (*n* = 2433:2433; mean 48 years; 72% women). Earnings data for 2001–2019 were retrieved from Statistics Sweden and analysed from 5 years before to 5 years after RA diagnosis.

**Results:**

No differences in average earnings were observed between siblings during the 5 years before diagnosis, but during the 5 years after diagnosis, patients with RA earned on average 5.4% less annually [–1430€ (95% CI –2130, −720)] than same-sexed siblings. The change in earnings for the subgroup diagnosed between 2006 and 2010 was –8.2% [–2020€ (95% CI –2930, –1120)] but for patients diagnosed between 2011 and 2017, there was no statistically significant change in earnings compared with siblings [–1.5%; –420€ (95% CI –1490, 640)]. Subgroup analyses demonstrated a more negative impact on earnings for older individuals and those with lower education level.

**Conclusion:**

RA diagnosis was associated with lower earnings in comparison with same-sex siblings, particularly for older individuals and those with lower education level. The negative impact of RA on earnings declined or disappeared over the study period.

Rheumatology key messages

- During 5 years after an RA diagnosis, patients’ earnings decreased by ∼5%.
- No association between RA diagnosis and future earnings was observed in patients diagnosed from 2011 to 2017.
- Older patients and those with lower education had earnings most negatively impacted by RA.

## Introduction

Treat-to-target strategies and the development of effective drugs since over the last decades have transformed RA management and improved patient outcomes [1]. The broader impact on patients’ lives, beyond clinical remission metrics, remains largely unknown. Specifically, the impact of RA on patients’ earnings in the modern era has not been studied.

Studies on the impact of RA on income have been conducted since the 1980s, but tend to be small (Supplementary Table S1, available at *Rheumatology* online). Household income has been shown to fall when someone in the household is diagnosed with RA [2–7]. These studies do not specifically investigate income earned from employment (i.e. earnings) but also include other sources of income, e.g. government transfers. Previous Swedish studies have indicated that patients with RA experience higher work loss starting ∼6 months before RA diagnosis, relative to matched general population comparators [8–12].

Most earlier studies of RA and earnings have lacked comparator groups (Supplementary Table S1, available at *Rheumatology* online), or used comparisons *vs* the general population. The former makes it difficult to draw conclusions and the latter tend to ignore underlying genetic and environmental factors among patients with RA [13].

The aim of this study was, therefore, to investigate the trajectory of earnings for patients in relation to RA diagnosis compared with same-sex siblings, overall as well as by calendar period of diagnosis. As a secondary outcome, we investigated the trajectory of disposable income (income from all sources minus income taxes).

## Methods

We performed a matched observational cohort study using prospectively collected data from Swedish registers. We used the unique Swedish personal identity number to link data from multiple nationwide registers. The Swedish Ethical Review Authority granted ethical approval for the data linkage without requiring informed consent.

Patients with incident RA were identified via the Swedish National Patient Register. Their same-sexed siblings were identified in the Multigeneration Register. Demographic data such as sex, date of birth, and migration dates were retrieved from the Total Population Register [14], and death dates from the Cause of Death Register. Baseline comorbidities (Supplementary Table S2, available at *Rheumatology* online) were retrieved from the National Patient Register, containing information on inpatient and outpatient care provided by Swedish hospitals and clinics. Comorbidity data were collected from 5 to 1 year before diagnosis, and dichotomously presented (yes/no) if present in these years [14].

### Participants

Patients aged 30–60 years with an incident RA diagnosis between 2006 and 2017 were included [14]. This age restriction typically corresponds to individuals who are in the prime of their working lives, with potentially higher income and greater financial responsibilities, and until 2020, the earliest age at which residents in Sweden could receive a public pension was 61 years.

Incident RA was defined based on a first-ever outpatient RA diagnosis (the index date), a second RA diagnosis within 1 year (inpatient or outpatient), at least 1 visit with the diagnosis at a rheumatology or internal medicine department and no history of any disease-modifying anti-rheumatic drugs in the 6 months before the first diagnosis (to prevent inclusion of prevalent cases). This definition has been robust in previous studies of RA in Sweden [15].

To be included, patients with RA were required to have a same-sexed sibling who was between 30 and 60 years at the index date and born no >5 years apart. If a patient with RA had several same-sex siblings, the sibling closest in age was selected. Siblings could not have a diagnosis of RA before the index date and were censored if they received a diagnosis during follow-up (flow chart in Supplementary Table S3, available at *Rheumatology* online).

### Outcome

The primary outcome was annual taxable earnings from the Longitudinal Integrated Database for Health Insurance and Labor Market Studies (LISA; data from 2001 to 2019, ensuring at least 2 years potential follow-up for all participants) [16], which covers the adult population in Sweden. The data originate from employer reports to the Swedish Tax Agency listing the taxable earned gross income in a calendar year (pension payments not included).

Annual disposable income was analysed as a secondary outcome. Disposable income is defined as total individual income, including capital income and transfers (e.g. child and sickness benefits), minus individual income taxes. Both taxable earnings and disposable income were included, as the former better reflects work ability and the latter the participant’s living standards. Earnings and income data were adjusted for annual inflation using the Swedish consumer price index and converted to 2019 Euros.

### Follow-up

To analyse the change in earnings after an RA diagnosis, we collected earnings data from 5 years before to 5 years after the year of diagnosis. Participants were followed until death (RA 2.6%; siblings 2.0%), emigration (RA 0.7%; siblings 0.7%) or end of follow-up (RA 96.7%; siblings 97.0%), whichever came first. Participants were censored for emigration or death at the year of the event.

### Statistical analyses

Prevalence and means with standard deviations were used to describe baseline variables. Annual mean differences in earnings and disposable income between patients with RA and their siblings were estimated by multiple regression adjusting for age, age squared, marital status and calendar year.

Changes in earnings and disposable income before and after RA diagnosis between patients with RA and their siblings were also assessed with difference-in-difference regression [17, 18], comparing the change over time among patients *vs* the change over time among siblings. The method was implemented as an interaction term between time (a dummy variable for before and after RA onset) and exposure (a dummy variable for RA *vs* sibling) in a regression model with sibling fixed effects (using variation within sibling pairs only) with additional covariates age, age squared, marital status and dummy variables for calendar year. The period from 5 to 1 year before diagnosis was considered before RA and the period from 1 to 5 years after diagnosis was considered after RA diagnosis. Standard errors were clustered at the sibling level to account for correlation of observations from the same sibling pairs over time.

#### Subgroup analyses

We also performed subgroup analyses by sex, median age at diagnosis (30–48 *vs* 49–60 years), education (no university *vs* university education; restricted to patient–sibling pairs with the same level of education) and median diagnosis year (2006–2010 *vs* 2011–2017). Subgroup differences were assessed with a triple interaction term for group membership, time, and exposure.

#### Sensitivity analysis

In sensitivity analysis, earnings data were winsorized (capped) at the 1st and 99th percentile per calendar year, replacing the smallest (<1st) and largest (>99th) earnings values with the 1st and 99th percentile, to reduce the effect of outliers.

## Results

### Participant characteristics

Among the 2433 patients with RA and their matched same-sex siblings, the mean age was 48 years, and 72% were women (Table 1). Patients with RA and siblings had similar education level. In the years before diagnosis (not including the 12 months before diagnosis), patients were more likely than their siblings to have had at least one visit associated with diseases of the musculoskeletal system and connective tissue.

**Table 1.** Participant characteristics at baseline

	Patients with RA (*n* = 2433), *N* (%)	Same-sex siblings (*n* = 2433), *N* (%)
Women	1750 (71.9)	1750 (71.9)
Age at diagnosis/index date		
Age (years), mean (SD)	47.9 (7.8)	47.8 (7.8)
Age (years), median (p25–p75)	49.4 (42.1-54.4)	49.2 (42.0-54.3)
Education		
Primary school	343 (14.1)	313 (12.9)
High school	1285 (52.8)	1281 (52.7)
University	803 (33.0)	833 (34.2)
*Education Missing*	*2 (0.1)*	*6 (0.2)*
Comorbidities^a^		
Cardiovascular disease	201 (8.3)	179 (7.4)
Psychiatric disorder	143 (5.9)	186 (7.6)
Substance use disorder	34 (1.4)	41 (1.7)
Musculoskeletal disorder (any)	607 (24.9)	399 (16.4)
Musculoskeletal disorder (inpatient)	76 (3.1)	66 (2.7)

a Retrieved from the National Patient Register, including inpatient and non-primary outpatient care, from 5 years before to 1 year before RA diagnosis.

### Main analyses

**Figure 1.**
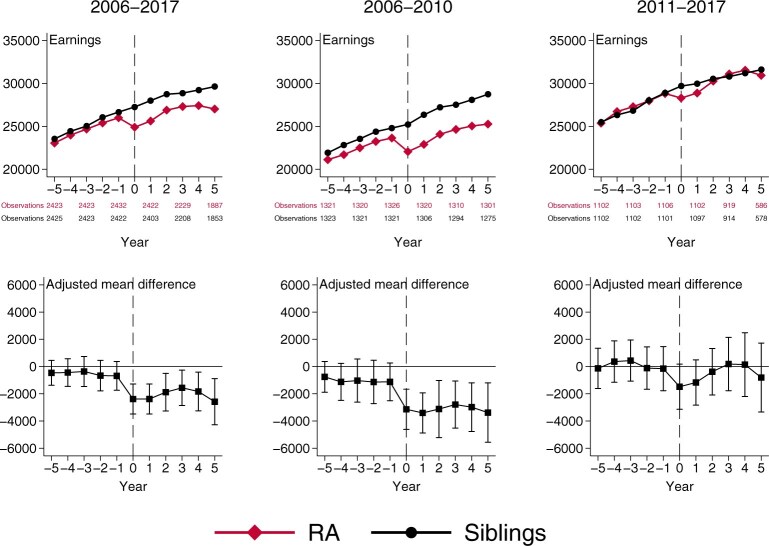
Earnings of patients with RA and their same-sex siblings by calendar period of diagnosis (top panels) and the adjusted mean differences (bottom panels)

After adjustment, no difference between patients with RA *vs* their same-sexed siblings in annual earnings was observed during any of the years before the year of diagnosis (Fig. 1). In the year of diagnosis, patients with RA earned less than their siblings, and this difference remained during all 5 years of follow-up. The lower earnings after diagnosis for patients with RA *vs* siblings was more pronounced at the 25th percentile (the lower end of the distribution) compared with the 50th and the 75th percentile (the upper end of the distribution; Supplementary Fig. S1, available at *Rheumatology* online). There was no difference in disposable income between patients with RA and their siblings, neither before nor after RA diagnosis (Supplementary Fig. S2, available at *Rheumatology* online).

The difference-in-difference estimate showed that after RA diagnosis, patients earned on average 5.4% less [–1430€ (95% CI –2130, –720)] per year than same-sex siblings (Supplementary Table S4, available at *Rheumatology* online). The estimate for disposable income was 0.7% less [–180€ (95% CI –1070, 710)] and not statistically significant.

### Subgroup analyses

Patients diagnosed between 2006 and 2010 had lower earnings after RA diagnosis [–8.2%; –2020€ (95% CI –2930, –1120)], while patients diagnosed after 2010 showed no difference *vs* their siblings [–1.5% or –420€ (95% CI –1490, 640); *P*_interaction_ = 0.026; Fig. 1, Supplementary Tables S4 and S6, available at *Rheumatology* online].

There was no statistically significant effect modification by sex, age at diagnosis or education level regarding earnings after RA diagnosis (Supplementary Tables S4 and S6, Supplementary Figs S3–S5, available at *Rheumatology* online).

### Sensitivity analyses

When earnings were winsorized, patients with RA had 4.9% lower earnings after diagnosis [difference-in-difference estimate –1300€ (95% CI –1890, –710); Supplementary Table S5, available at *Rheumatology* online]. In patients diagnosed between 2011 and 2017, the decline was 1.7% [–480€ (95% CI –1450, 490)]. After winsorization, effect modification was observed by age at diagnosis (*P*_interaction_ = 0.007), education level (*P*_interaction_ = 0.026) and diagnosis period (*P*_interaction_ = 0.035; Supplementary Tables S5 and S6, available at *Rheumatology* online).

## Discussion

In our study, patients with RA experienced a decrease in taxable earnings of 5.4% after diagnosis, which was sustained for at least 5 years. This drop was driven by patients diagnosed between 2006 and 2010, while patients diagnosed between 2011 and 2017 did not display a difference in average earnings compared with their same-sexed siblings.

Previous studies have found that RA can negatively affect income and productivity [2–4, 19]. Studies from the 1980s, 1990s and early 2000s typically lack appropriate comparators, are small and use self-reported outcome data. Our study is the first of its kind using large-scale register data comparing patients with same-sex siblings. It is also the first study to show a calendar period effect with lower earnings after diagnosis in the beginning of the biologic era and no difference among patients diagnosed between 2011 and 2017.

There were subgroup variations when assessing the association between RA and subsequent earnings. Older individuals and those with lower education levels experienced significantly lower earnings after their RA diagnosis relative to same-sex siblings, whereas younger individuals and those with higher education did not (or at least not to the same degree).

Furthermore, patients diagnosed between 2006 and 2010 experienced a statistically significant decline in earnings compared with their siblings. In contrast, patients diagnosed between 2011 and 2017 did not differ in post-diagnosis earnings compared with same-sex siblings, and the point estimate was near null, though negative. Our interpretation is that, despite the introduction of biologic therapies in the late 1990s and early 2000s, the positive treatment effect on earnings may not have been fully affected until about a decade later, at least not in Swedish patients. A contributing factor may be that the median disease duration at biologic treatment initiation fell for patients with RA during the first 10 years after the introduction of biologics [20]. External factors such as the business cycle and institutional changes over time may also have played a role, but affect both patients and sibling controls simultaneously.

The main strength of this study is the use of nationwide registers, which provided objectively assessed and virtually complete earnings data on a large number of patients. Furthermore, we had data from 5 years before to 5 years after diagnosis, enabling assessment of pre-diagnostic similarity between patients and siblings. The use of siblings as a control group also reduced the influence of changes in the social security system on our findings since potential changes likely affected patients and siblings in a similar way.

A potential limitation is the generalizability of our findings. In a society with a generous welfare system, the reduction in income is partially buffered through government transfers. Our analysis demonstrated that patients experienced reduced earning capacity in Sweden before 2011 but no reduction in disposable income. This shows the importance of being able to separate earnings from disposable income, especially in countries with generous welfare systems. Another limitation was the requirement for patients to have a same-sex sibling.

In conclusion, a diagnosis of RA had a negative impact on patients’ annual taxable earnings trajectories over 5 years but little to no impact on disposable income. The difference in earnings between patients and siblings was driven by patients diagnosed before 2011, whereas patients diagnosed during the last decade did not differ from their siblings in terms of earnings after diagnosis.

## Supplementary Material

keae535_Supplementary_Data

## Data Availability

Data may be obtained from a third party and are not publicly available. The study data forms part of a register linkage performed by Karolinska Institutet, and for which further sharing of the data is limited by legal restrictions.

## References

[1] Josef SS , Daniel A, Johannes WJB et al Treating rheumatoid arthritis to target: recommendations of an international task force. Ann Rheum Dis 2010;69:631. 20215140 10.1136/ard.2009.123919 PMC3015099

[2] Wolfe F , Michaud K, Choi HK, Williams R. Household income and earnings losses among 6,396 persons with rheumatoid arthritis. J Rheumatol 2005;32:1875–83. 16206340

[3] Fox SR , Masi AT, Robinson H, Jacob DL, Kaplan SB. Earnings of early diagnosed arthritis patients and matched controls. J Chronic Dis 1976;29:469–78. 939802 10.1016/0021-9681(76)90087-4

[4] Sullivan PW , Ghushchyan V, Huang X, Globe D. Influence of rheumatoid arthritis on employment, function, and productivity in a nationally representative sample in the United States. J Rheumatol 2010;37:544–9. 20080920 10.3899/jrheum.081306

[5] Vu M , Carvalho N, Clarke PM, Buchbinder R, Tran-Duy A. Impact of comorbid conditions on healthcare expenditure and work-related outcomes in patients with rheumatoid arthritis. J Rheumatol 2021;48:1221–9. 33323533 10.3899/jrheum.200231

[6] Walker N , Michaud K, Wolfe F. Work limitations among working persons with rheumatoid arthritis: results, reliability, and validity of the work limitations questionnaire in 836 patients. J Rheumatol 2005;32:1006–12. 15940759

[7] Shanahan EM , Smith MD, Roberts-Thomson L, Esterman A, Ahern MJ. The effect of rheumatoid arthritis on personal income in Australia. Intern Med J 2008;38:575–9. 18028367 10.1111/j.1445-5994.2007.01546.x

[8] Neovius M , Simard JF, Klareskog L, Askling J, Group AS; ARTIS Study Group. Sick leave and disability pension before and after initiation of antirheumatic therapies in clinical practice. Ann Rheum Dis 2011;70:1407–14. 21518724 10.1136/ard.2010.144139

[9] Neovius M , Simard JF, Askling J; ARTIS Study Group. How large are the productivity losses in contemporary patients with RA, and how soon in relation to diagnosis do they develop? Ann Rheum Dis 2011;70:1010–5. 21406455 10.1136/ard.2010.136812

[10] Sokka T , Kautiainen H, Möttönen T, Hannonen P. Work disability in rheumatoid arthritis 10 years after the diagnosis. J Rheumatol 1999;26:1681–5. 10451062

[11] Eriksson JK , Neovius M, Bratt J et al Biological vs. conventional combination treatment and work loss in early rheumatoid arthritis: a randomized trial. JAMA Intern Med 2013;173:1407–14. 23817631 10.1001/jamainternmed.2013.7801

[12] Olofsson T , Petersson IF, Eriksson JK et al Predictors of work disability during the first 3 years after diagnosis in a national rheumatoid arthritis inception cohort. Ann Rheum Dis 2014;73:845–53. 23520035 10.1136/annrheumdis-2012-202911

[13] Frisell T , Oberg S, Kuja-Halkola R, Sjolander A. Sibling comparison designs: bias from non-shared confounders and measurement error. Epidemiology 2012;23:713–20. 22781362 10.1097/EDE.0b013e31825fa230

[14] Ludvigsson JF , Almqvist C, Bonamy AK et al Registers of the Swedish total population and their use in medical research. Eur J Epidemiol 2016;31:125–36. 26769609 10.1007/s10654-016-0117-y

[15] Eriksson JK , Neovius M, Ernestam S et al Incidence of rheumatoid arthritis in Sweden: a nationwide population-based assessment of incidence, its determinants, and treatment penetration. Arthritis Care Res (Hoboken) 2013;65:870–8. 23281173 10.1002/acr.21900

[16] Ludvigsson JF , Svedberg P, Olen O, Bruze G, Neovius M. The longitudinal integrated database for health insurance and labour market studies (LISA) and its use in medical research. Eur J Epidemiol 2019;34:423–37. 30929112 10.1007/s10654-019-00511-8 PMC6451717

[17] Wing C , Simon K, Bello-Gomez RA. Designing difference in difference studies: best practices for public health policy research. Annu Rev Public Health 2018;39:453–69. 29328877 10.1146/annurev-publhealth-040617-013507

[18] Caniglia EC , Murray EJ. Difference-in-difference in the time of cholera: a gentle introduction for epidemiologists. Curr Epidemiol Rep 2020;7:203–11. 33791189 10.1007/s40471-020-00245-2 PMC8006863

[19] Meenan RF , Yelin EH, Nevitt M, Epstein WV. The impact of chronic disease: a sociomedical profile of rheumatoid arthritis. Arthritis Rheum 1981;24:544–9. 7213432 10.1002/art.1780240315

[20] Simard JF , Arkema EV, Sundstrom A et al Ten years with biologics: to whom do data on effectiveness and safety apply? Rheumatology (Oxford) 2011;50:204–13. 21084326 10.1093/rheumatology/keq326

