# Correction to: Impact of incident rheumatoid arthritis on earnings: a nationwide sibling comparison study

**DOI:** 10.1093/rheumatology/keae712

**Published:** 2025-01-06

This is a correction to: Heather Miller, Martin Neovius, Johan Askling, Gustaf Bruze, Impact of incident rheumatoid arthritis on earnings: a nationwide sibling comparison study, *Rheumatology*, 2024, keae535, https://doi.org/10.1093/rheumatology/keae535

Acknowledgement of funding from the Swedish Rheumatism Association was omitted from the originally published version and has been added.

