# Supplementary material for: Impact of incident rheumatoid arthritis on earnings: a nationwide sibling comparison study
Source: Rheumatology (Oxford). 2024 Oct 16;64(6):3879–83. doi: 10.1093/rheumatology/keae535 (PMC12107035; doi:10.1093/rheumatology/keae535)
Supplement: keae535_Supplementary_Data [file keae535_supplementary_data.pdf]

Supplementary Appendix

Impact of incident rheumatoid arthritis on earnings:

a nationwide sibling comparison study

Heather Miller, Martin Neovius, Johan Askling, Gustaf Bruze

Clinical Epidemiology Division, Department of Medicine Solna,
Karolinska Institutet, Stockholm, Sweden

Table of Contents

**Object**	**Page**	**Description**
Table S1	16	Evidence table of earlier studies
Table S2	17	Data sources for baseline data
Table S3	18	Flow chart
Table S4	19	Difference-in-difference estimates (Euros) for the main analysis.
Table S5	20	Difference-in-difference estimates (Euros) with winsorized data
Table S6	21	Subgroup analysis
Figure S1	22	Earnings (left) and disposable income (right) of patients with RA (n=2433) and their same-sex siblings (n=2433) and adjusted mean annual differences (bottom).
Figure S2	23	Percentiles of the earnings distribution (25th, 50th, and 75th percentiles) for patients with RA and their same-sex siblings.
Figure S3	24	Earnings stratified by sex
Figure S4	25	Earnings stratified by age at diagnosis
Figure S5	26	Earnings stratified by education

Table S1. Evidence table of studies investigating income and earnings in patients with RA.

Study	Year	Participant Groups	N	Outcome(s)	Findings
Walker N, Michaud K, Wolfe F. Work limitations among working persons with rheumatoid arthritis: results, reliability, and validity of the work limitations questionnaire in 836 patients. J Rheumatol. 2005 Jun;32(6):1006-12.	2001-2002	RA patients (United States)	836	- Self-reported work disability<br>- Self-reported disability benefits<br>- Self-reported earnings	- Persons with RA tend to select jobs that they can do with their RA limitations<br>- 27.9% of patients aged < or = 65 years considered themselves disabled after 14.6 years of RA<br>- 8.8% received disability benefits<br>- Annual earnings losses ranged between USD 2,319 and USD 3,407 by the CPS and internal method (preferred), with losses of 9.3% and 10.9%.
Wolfe F, Michaud K, Choi HK, Williams R. Household income and earnings losses among 6,396 persons with rheumatoid arthritis. J Rheumatol. 2005 Oct;32(10):1875-83.	2001-2002	RA patients and members of the same household (United States)	6,649	- Self-reported household income<br>- Expected earnings from Current Population Survey (CPS) and O*NET (Occupational Information Network) data	- The overall impact of RA on household income is USD 6,287 (11.8%)<br>- Earnings and household income are dependent on functional status, education, age, ethnicity, and marital status.
Shanahan EM, Smith MD, Roberts-Thomson L, Esterman A, Ahern MJ. The effect of rheumatoid arthritis on personal income in Australia. Intern Med J. 2008 Jul;38(7):575-9.	2003-2004	RA patients (Australia)	479	- Self-reported income<br>- Self-reported income loss	- When standardized, the income of our cohort was 66% that of the average income of the Australian population.<br>- 1/3 relied principally on social security system for their income
Fox SR, Masi A, Robinson H, Jacob D, Kaplan SB. Earnings of early diagnosed arthritis patients and matched controls.. Journal of Chronic Diseases, Volume 29, Issue 7, 1976, Pages 469-478.	1968-1972	RA patients & matched general population controls (matched by baseline earnings) (United States)	23	- Self-reported earnings	- RA patients and matched controls had the same income before diagnosis but were significantly less likely to increase earnings after diagnosis
Vu M, Carvalho N, Clarke PM, Buchbinder R, Tran-Duy A. Impact of Comorbid Conditions on Healthcare Expenditure and Work-related Outcomes in Patients With Rheumatoid Arthritis. J Rheumatol. 2021 Aug;48(8):1221-1229.	2006-2015	RA patients (United States)	4967	- Survey data (self-reported)	- Comorbid conditions in patients with RA were associated with higher annual healthcare expenditure, lower likelihood of employment, higher rates of absenteeism, and lower income.

Table S2. Data sources for baseline data

Baseline data	Source	ICD 10 Code
Cardiovascular disease	National Patient Register	I00-I99 Disease of the circulatory system
Psychiatric disorder	National Patient Register	F00-F99 Mental and behavioral disorders
Substance abuse	National Patient Register	F10-F19 Mental and behavioural disorders due to psychoactive substance use
Musculoskeletal disorder	National Patient Register	M00-M99 Diseases of the Musculoskeletal System and Connective Tissue

Table S3. Flow chart

RA PATIENTS WITH SAME-SEX FULL SIBLINGS	
Patients with RA diagnosis in outpatient care 2006 or later (first diagnosis is index date)	64423
No prior RA diagnosis in inpatient care before index date	57687
A second RA diagnosis (outpatient or inpatient) within one year from index date	43581
First or second RA diagnosis by a specialist	38546
Age at index date between 30 and 60 years	15605
No DMARDs in Prescribed Drug Register during six months before index date	11521
No DMARDs in SRQ during six months before index date	11387
At least two years potential follow-up	9090
RA patients has at least one full sibling	6283
RA patients has at least one same-sex full sibling	4116
Sibling is alive at index date	4018
Sibling is between 30 and 60 years at index date	3358
Age difference between siblings is less than 5 years	2525
Sibling does not have RA diagnosis in Patient Register before index date	2467
Sibling has earnings data	2433

Table S4. Difference-in-difference estimates (Euros) for the main analysis.

<u>Earnings</u>	**Number of Observations** RA/Siblings	**Estimate**	**[95%CI]**	**P-Value**	**Percentage Change***
**Overall**	**2433/2433**	**-1430**	**[-2130; -720]**	**<0.001**	**-5.4**
Women	1750/1750	-1470	[-2150; -800]	<0.001	-6.0
Men	683/683	-1340	[-3170; 480]	0.148	-4.1
Younger (30-48y)	1180/1180	-660	[-1780; 470]	0.251	-2.5
Older (49-60y)	1253/1253	-1980	[-2820; -1150]	<0.001	-7.4
No university degree	1249/1249	-1920	[-2720; -1120]	<0.001	-8.5
University degree	1184/1184	-930	[-2110; 260]	0.126	-2.7
Diagnosed 2006-2010	1326/1326	-2020	[-2930; -1120]	<0.001	-8.2
Diagnosed 2011-2017	1107/1107	-420	[-1490; 640]	0.435	-1.5
<u>**Disposable Income**</u>					
**Overall**	**2433/2433**	**-180**	**[-1070; 710]**	**0.691**	**-0.7**
Women	1750/1750	-540	[-1100; 20]	0.060	-2.3
Men	683/683	770	[-2070; 3610]	0.595	2.6
Younger (30-48y)	1180/1180	260	[-1290; 1800]	0.745	1.0
Older (49-60y)	1253/1253	-600	[-1470; 260]	0.170	-2.4
No university degree	1249/1249	-780	[-1460; -110]	0.023	-3.5
University degree	1184/1184	450	[-1240; 2130]	0.604	1.5
Diagnosed 2006-2010	1326/1326	-780	[-1440; -130]	0.019	-3.3
Diagnosed 2011-2017	1107/1107	810	[-1150; 2760]	0.418	3.0

* Percentage change relative to earnings (or disposable income) of RA patients in the year before RA diagnosis.

Table S5. Difference-in-difference estimates (Euros) with winsorized data.

Earnings	Observations RA/siblings	Estimate	[95%CI]	P-value	Percentage change*
**Overall**	**2433/2433**	**-1300**	**[-1890; -710]**	**<0.001**	**-4.9**
Women	1750/1750	-1350	[-2000; -700]	<0.001	-5.5
Men	683/683	-1180	[-2450; 80]	0.067	-3.6
Younger (30-48y)	1253/1253	-400	[-1280; 470]	0.367	-1.5
Older (49-60y)	1180/1180	-1990	[-2780; -1200]	<0.001	-7.5
No university degree	1249/1249	-1950	[-2710; -1190]	<0.001	-8.6
University degree	1184/1184	-610	[-1520; 290]	0.184	-1.8
Diagnosed 2006-2010	1326/1326	-1790	[-2520; -1060]	<0.001	-7.2
Diagnosed 2011-2017	1107/1107	-480	[-1450; 490]	0.330	-1.7
**Disposable Income**					
**Overall**	**2433/2433**	**-280**	**[-640; 80]**	**0.125**	**-1.1**
Women	1750/1750	-270	[-670; 130]	0.182	-1.2
Men	683/683	-310	[-1080; 460]	0.427	-1.1
Younger (30-48y)	1180/1180	-10	[-540; 530]	0.985	-0.0
Older (49-60y)	1253/1253	-550	[-1040; -60]	0.028	-2.2
No university degree	1249/1249	-570	[-1020; -110]	0.015	-2.5
University degree	1184/1184	10	[-550; 580]	0.964	0.0
Diagnosed 2006-2010	1326/1326	-580	[-1000; -160]	0.007	-2.5
Diagnosed 2011-2017	1107/1107	200	[-430; 820]	0.534	0.7

* Percentage change relative to earnings/disposable income of RA patients in the year before RA diagnosis.

Table S6. Subgroup differences assessed by use of interaction terms.

NON-WINSORIZED DATA			
**Earnings**	**Estimate**	**[95%CI]**	**P-value**
Men vs Women	160	[-1780; 2100]	0.872
30-48y vs 49-60y at diagnosis	1360	[-50; 2770]	0.060
No university vs university education	-1000	[-2430; 430]	0.170
Diagnosed 2006-2010 vs 2011-2017	-1590	[-3000; -190]	0.026
**Disposable Income**			
Men vs Women	1330	[-1540; 4200]	0.364
30-48y vs 49-60y at diagnosis	-850	[-2630; 920]	0.346
No university vs university education	-1230	[-3040; 580]	0.184
Diagnosed 2006-2010 vs 2011-2017	-1590	[-3650; 460]	0.129
**WINSORIZED DATA**			
**Earnings**	**Estimate**	**[95%CI]**	**P-value**
Men vs Women	200	[-1220; 1620]	0.783
30-48y vs 49-60y at diagnosis	1630	[450; 2810]	0.007
No university vs university education	-1340	[-2530; -160]	0.026
Diagnosed 2006-2010 vs 2011-2017	-1300	[-2520; -90]	0.035
**Disposable Income**			
Men vs Women	-10	[-880; 860]	0.981
30-48y vs 49-60y at diagnosis	560	[-160; 1280]	0.129
No university vs university education	-590	[-1310; 140]	0.112
Diagnosed 2006-2010 vs 2011-2017	-780	[-1530; -30]	0.043

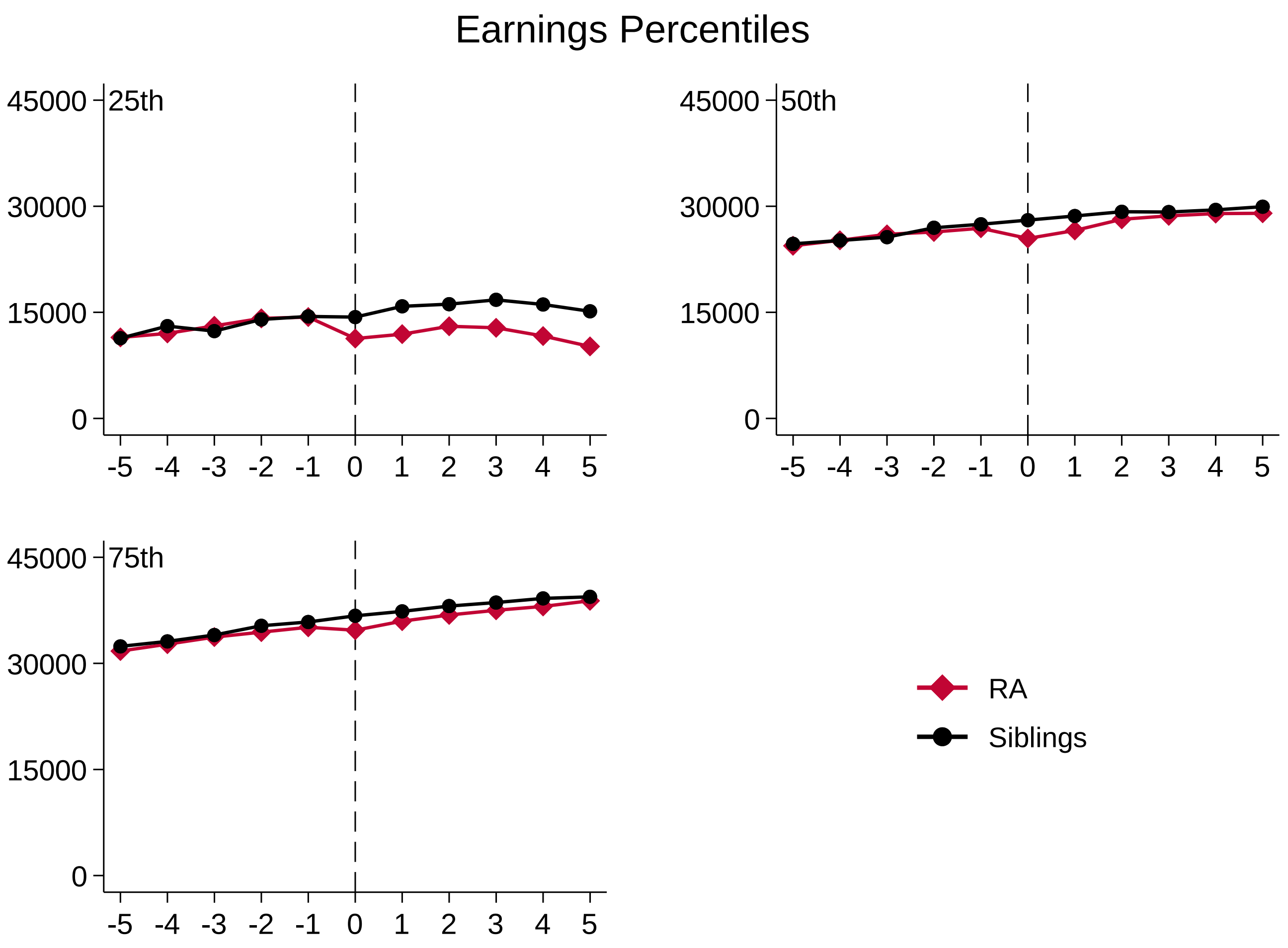

Figure S1. Percentiles of the earnings distribution (25th, 50th, and 75th percentiles) for patients with RA and their same-sex siblings.

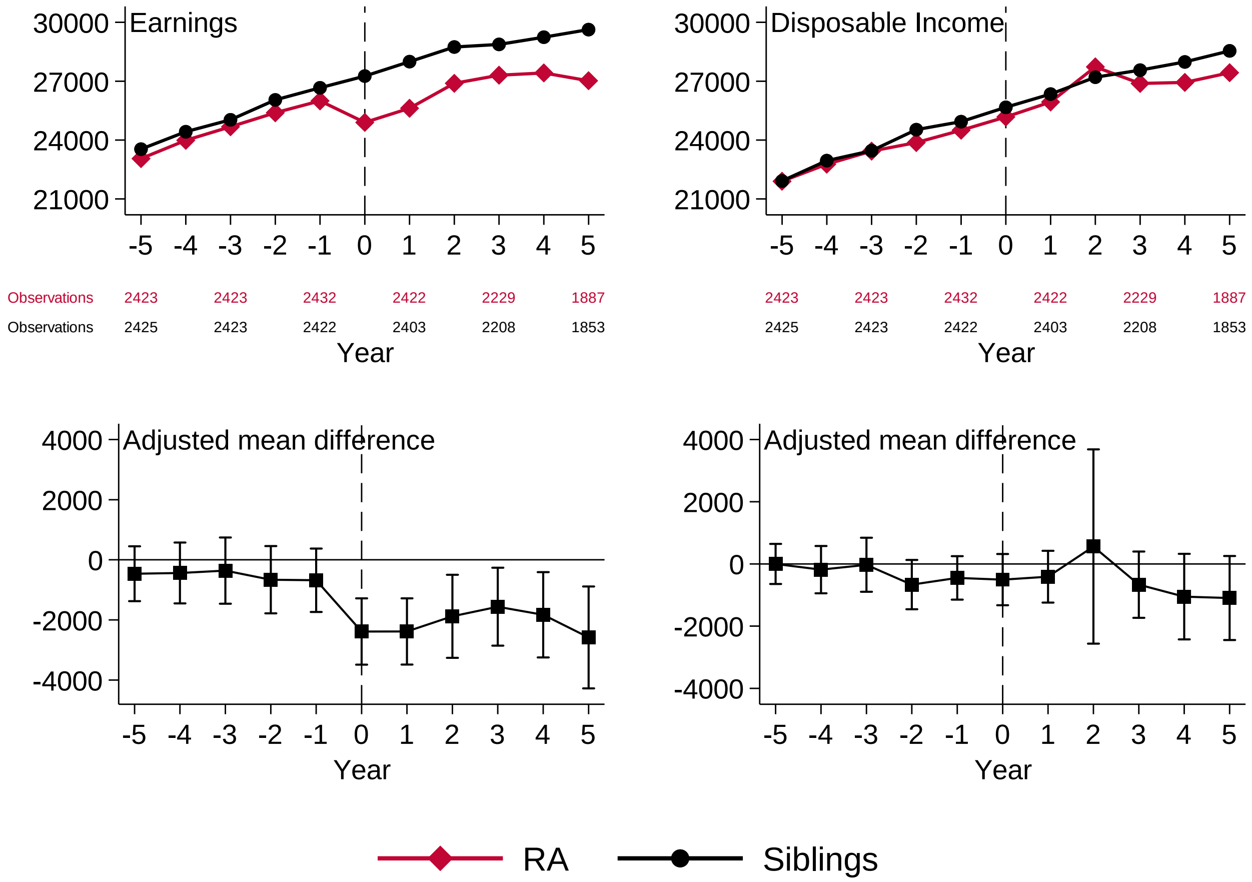

Figure S2. Earnings (left) and disposable income (right) of patients with RA (n=2433) and their same-sex siblings (n=2433) and adjusted mean annual differences (bottom).

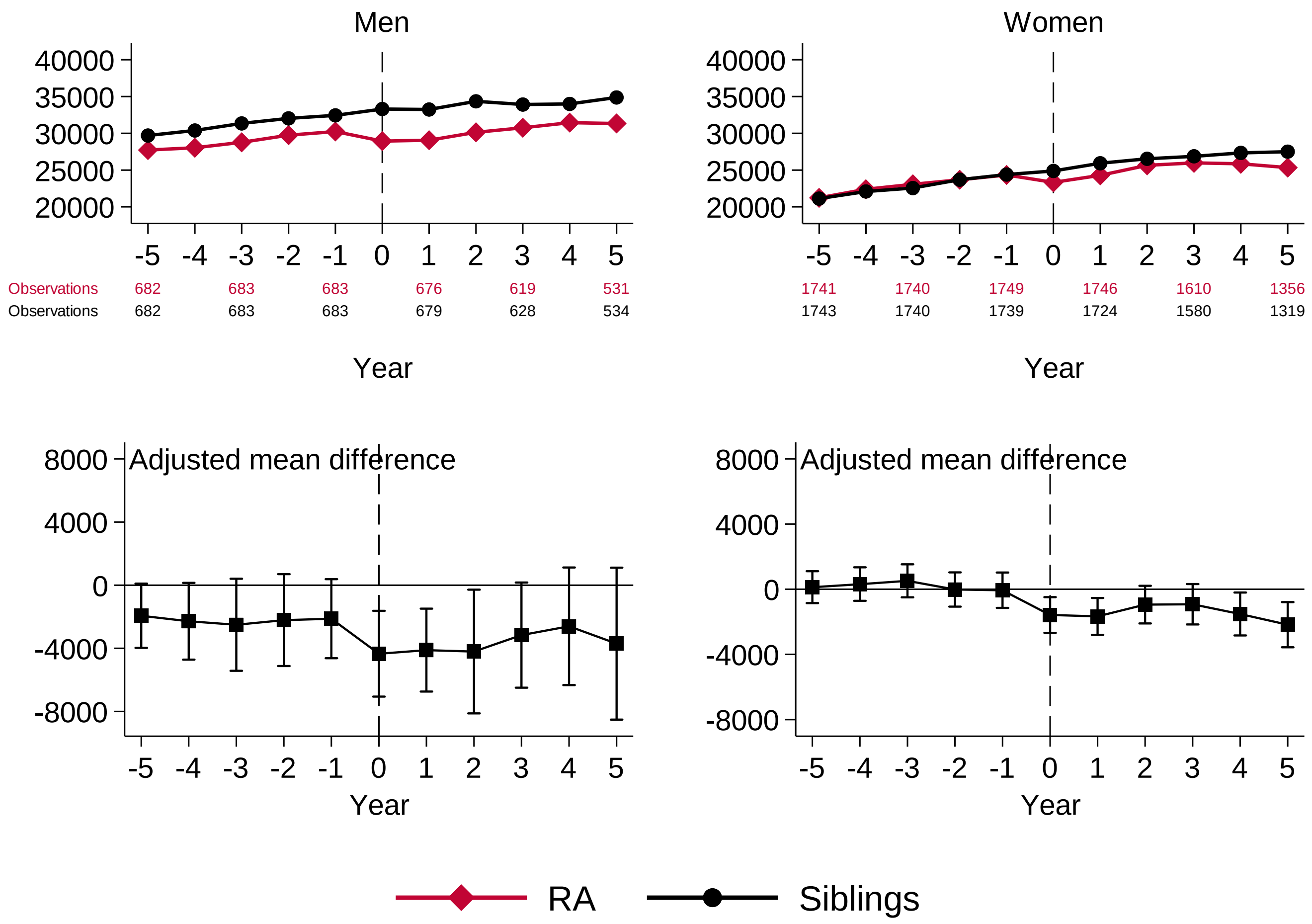

Figure S3. Earnings of patients with RA and their same-sex siblings stratified by sex.

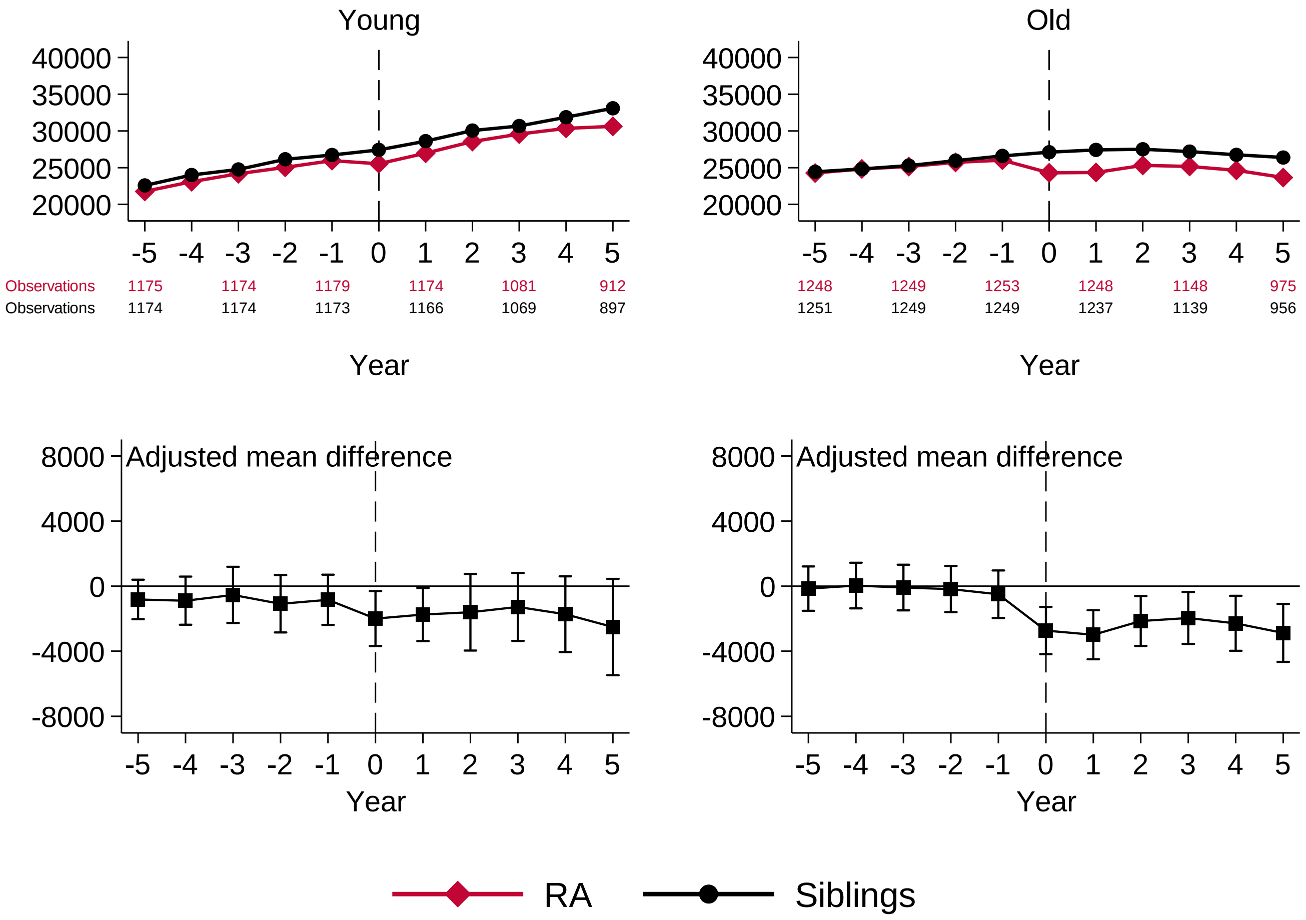

Figure S4. Earnings of patients with RA and their same-sex siblings stratified by median age at diagnosis (30-48y versus 49-60y).

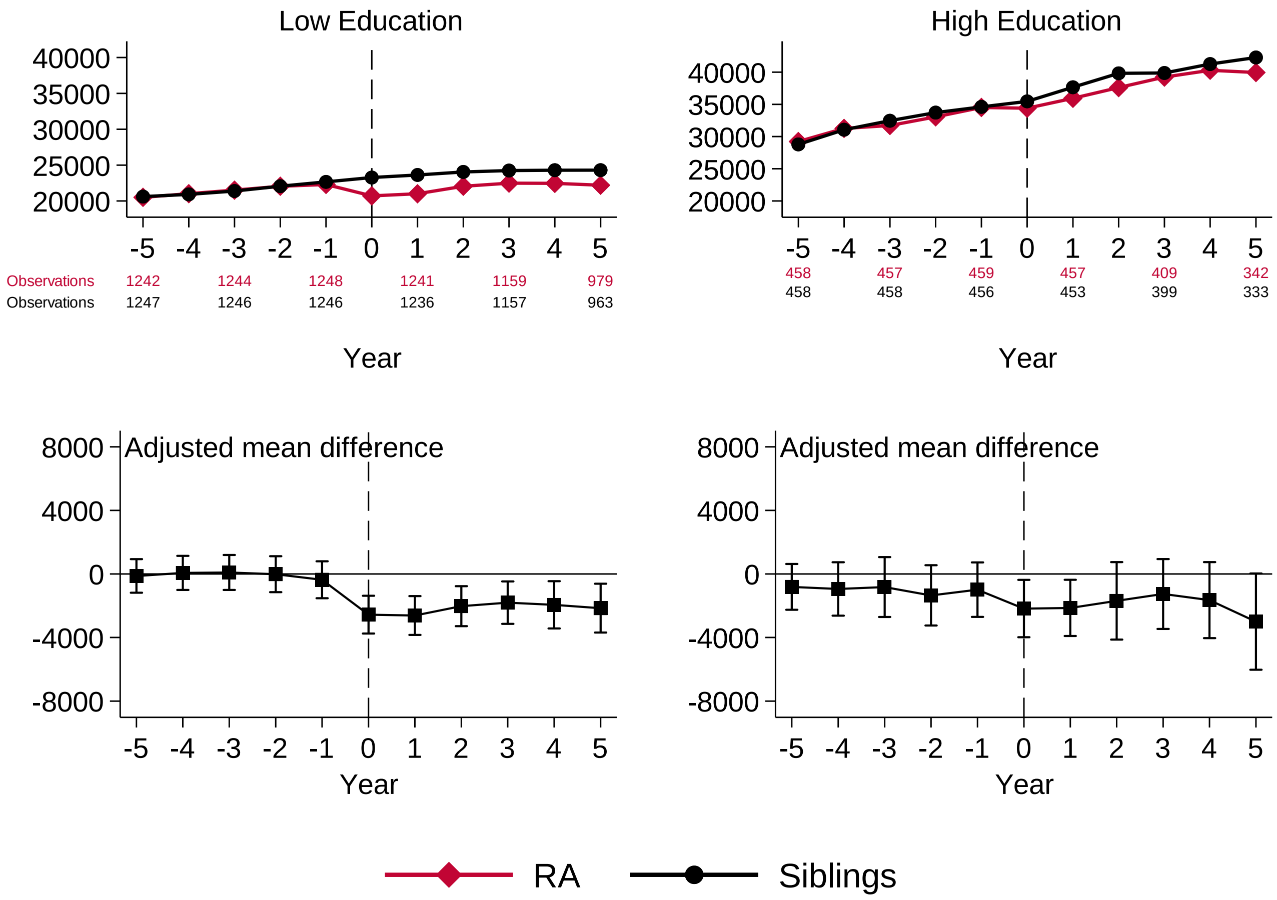

Figure S5. Earnings of patients with RA and their same-sex siblings stratified by no university degree (low education) versus university degree (high education).
